# Evaluating the Informed Consent Objective Structured Clinical Exam (OSCE) Using a Minimum Standard Assessment Tool During the Transition-to-Residency Program

**DOI:** 10.7759/cureus.109515

**Published:** 2026-05-23

**Authors:** Catherine Chen, Amanda Esposito, Elizabeth Goodman, Neha Pidatala, Raman Bhalla

**Affiliations:** 1 Hospital Medicine, Robert Wood Johnson University Hospital, New Brunswick, USA; 2 Emergency Medicine, Robert Wood Johnson University Hospital, New Brunswick, USA; 3 Pediatrics, Rutgers Robert Wood Johnson Medical School, New Brunswick, USA; 4 Consulting, Covera Health, New York, USA; 5 General Internal Medicine, Robert Wood Johnson University Hospital, New Brunswick, USA

**Keywords:** informed consent, medical education research, objective structured clinical exam (osce), patient centered care, quality management in medical education and health care services

## Abstract

Objective: To evaluate the feasibility and descriptive performance of a modified minimum-standard informed consent (IC) assessment tool when implemented during a transition-to-residency IC objective structured clinical exam (OSCE).

Study setting and design: We conducted a single-center retrospective cohort analysis of IC OSCE recordings from fourth-year medical students enrolled in a transition-to-residency course in Academic Year (AY) 2023 and AY2024. Facilitators scored recordings using a modified Spatz minimum-standard tool.

Minimum standard was considered addressing either quantitative or qualitative risk in addition to the domains of “What,” “Why,” “How,” “Benefits,” “Alternatives,” and “Timeframe.” Each domain was worth one point for a total minimum standard score of seven.

Data sources and analytic sample: Primary data was collected during March/April 2023 (AY2023) and March/April 2024 (AY2024) using the modified Spatz assessment tool completed by facilitators while watching IC OSCE recordings.

Principal findings: In 2023 and 2024, 158 (98.75%) and 155 (96.87%) OSCEs were graded, respectively. A greater proportion of AY2024 students met the minimum standard compared with AY2023 students (87.1% vs 51.9%, p < 0.001). Higher AY2024 domain-level performance was observed for “Alternatives” and “Timeframe” as well as in “Quantitative Risk.” A high proportion of students met expectations on the “What,” “Why,” “How,” and “Benefits” sections in both years. Students in specialty subgroups with procedural predominance maintained high performance. Non-procedural specialty students improved significantly from AY2023 to AY2024.

Conclusions: The modified Spatz assessment tool was feasible to implement in a transition-to-residency IC OSCE and identified specific consent-content domains in which students did and did not meet a minimum standard.

## Introduction

The American Association of Medical Colleges (AAMC) published guidelines on 13 entrustable professional activities (EPA) in 2014, outlining specific skills all medical students should be proficient in as they begin residency, regardless of their chosen specialty [1]. The ability to independently obtain appropriate informed consent (IC) is one of those 13 required EPAs [1]. The ability to obtain consent is fundamental in building successful physician-patient relationships, supporting patient autonomy, and ensuring patients make informed care decisions [2]. 

Throughout the clinical years, students repeatedly observe the structured dialogue of IC across various specialties [3]. However, multiple studies have shown that new interns “are not confident or competent in their ability to perform an appropriate informed consent discussion”, despite this being identified as a vital skill upon completion of medical school by the AAMC [4,5]. During a transition-to-residency (TTR) course, occurring just prior to the completion of the medical school curriculum, we implemented a version of the eight-domain IC abstraction tool created by Spatz et al. [6] to assess whether students met the minimum standard of IC [7] during an objective structured clinical exam (OSCE). The primary aim of this study was to evaluate the feasibility and descriptive performance of this modified minimum-standard tool in a TTR OSCE setting. A secondary exploratory aim was to describe year-to-year cohort differences in domain-level and overall minimum-standard performance.

## Materials and methods

OSCE description

The IC OSCE was administered during the TTR course to 160 fourth-year medical students from each graduating class of academic year (AY) 2023 and AY 2024. The OSCE was designed to evaluate students’ proficiency in obtaining IC for transfusion of blood products in a simulated clinical scenario with a standardized patient (SP). The blood transfusion consent scenario, SP case materials, video-recording process, rubric domains, and minimum-standard scoring threshold were maintained across the two study years.

Each SP encounter was recorded for subsequent review and evaluation by TTR facilitators. The IC OSCE was completed by all students regardless of their post-graduate specialty. The study did not include an independent external measure of IC competence outside the OSCE encounter.

Minimum consent tool

The assessment tool used in this study was based on the original minimum-standard rubric developed by Spatz et al. [6], and adapted by Marwaha et al. (Appendix) [7].

The tool included eight domains: “What,” “Why,” “How,” “Benefits,” “Qualitative Risks,” “Quantitative Risks,” “Alternatives,” and “Timeframe.” Each domain was weighted 1 point on a binary scale (1 = addressed adequately, 0 = incomplete, inadequate, or inaccurate).

A score that met the minimum standard was defined as a total score of seven, with the requirement that students adequately address at least one of two risk domains in addition to all other domains. Feedback was provided to students during their OSCE review.

Data collection and grading

TTR facilitators watched video recordings of the student-SP interactions and graded accordingly using the minimum-standard tool on Redcap. Each recording was scored by a single facilitator as part of the course evaluation process. Prior to the IC OSCEs in AY2023, facilitators were introduced to the rubric and an abstraction training manual was provided and reviewed. In AY2024, the assessment tool was disseminated to grading facilitators without additional formal training. 

Statistical analysis

Analyses were performed using IBM SPSS v29.0.1.0 (IBM Corp., Armonk, NY, USA) software. Statistical significance for total score was assessed using Mann-Whitney U test. Two-tailed Fisher’s exact test was used for each IC domain and minimum standard, including specialty subgroup analysis. Significance was set at p <0.05. Descriptive statistics were performed for sub-questions in each domain.

Comparisons between AY2023 and AY2024 were interpreted as cohort comparisons under similar but not identical implementation conditions. Comparisons involving facilitator groups were considered exploratory comparisons of pass rates, not formal inter-rater reliability analyses.

## Results

Student characteristics

In 2023 and 2024, facilitators graded 158 (98.75% of all enrolled students) and 155 (96.87% of all enrolled students) OSCEs respectively during the TTR courses. Students were enrolled in specialty bootcamp subgroups based on their post-graduate specialty (emergency medicine, family medicine, internal medicine, obstetrics and gynecology (Ob-Gyn), pediatrics, psychiatry, and surgery).

IC domains

**Figure 1:**
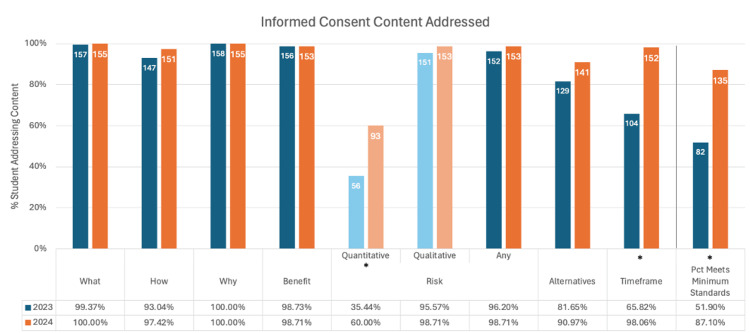
Informed consent domain scores for years 2023 and 2024. Statistical significance is indicated by (*).

A high proportion of students met expectations in the “What” (N = 157, 99.3% in 2023, N = 155, 100% in 2024), “How” (N = 147, 93.0% in 2023, and N = 151, 97.4% in 2024), “Why” (N = 158 in 2023, 155 in 2024, 100% both years), and “Benefits” (N = 156 in 2023, N = 153 in 2024, 98.7% both years) (Figure 1).

For students who did not receive points for “How,” the reasons were that the “Information about the procedure that does not explain HOW the procedure will be performed” (N = 8, 5% in 2023 and N = 12, 7.7% in 2024) and no description of the procedure was provided (N = 3, 1.9% in 2023, N=0 in 2024). For “Benefits”, the students in each year who did not receive points either only explained the “clinical rationale without referring to a positive effect” (N = 1 in both years, 0.63% in 2023, 0.65% in 2024) or only “state[d] possible benefits, not defining primary (intended) benefit(s)” (N = 1 also in both years).

Discussion of “Risk,” either “Quantitative” or “Qualitative,” was also high at 96.2% (N = 152) and 98.7% (N = 153) for AY2023 and AY2024, respectively. Statistically significant improvement was seen in “Quantitative Risk” with increase from 35.4% (N = 56) to 60% (N = 93) in subsequent years (p <0.001). For students who did not receive points for “Quantitative Risk,” the most common reason was that students gave only a “generic statement that the risks were discussed with the patient.”

“Alternatives” were discussed by 81.7% (N = 129) and 91.0% (N = 141) of students in AY2023 and AY2024, respectively, which was statistically significant with p = 0.021. For students who did not receive points for “Alternatives” the most common reason was that it was “not provided” or a “generic statement” was mentioned about alternatives and the “patient’s right to forgo the procedure.” 

Discussion of procedural “Timeframe” was more frequently observed in AY2024 compared to AY2023 (98.1% (N = 152) vs 65.8% (N = 104), p<0.001). For students who did not receive points for “Timeframe,” it was most commonly due to “not provided” or “non-descript terms were used.”

Overall, 51.9% (N = 82) of AY2023 students and 87.1% (N = 135) of AY2024 students met the minimum standard (p < 0.001).

Specialty subgroup analysis

Significant improvements were seen in all subgroups except for psychiatry and surgery (Table 1).

**Table 1:** Specialty Subgroup Analysis for individual and clustered specialties by procedural intensiveness * Significant decrease in meeting the minimum standard.

Specialty	2023 Meets Minimum Standard n/N (%)	2024 Meets Minimum Standard n/N (%)	p-value
Non-Procedural Specialties	41/106 (38.7%)	83/94 (88.3%)	<0.001
Family Medicine	8/14 (57.1%)	15/16 (93.8%)	0.031
Internal Medicine	17/60 (28.3%)	47/54 (87.0%)	<0.001
Pediatrics	5/18 (27.8%)	14/16 (87.5%)	<0.001
Psychiatry	11/14 (78.6%)	7/8 (87.5%)	1.000
Procedural Specialties	41/52 (78.8%)	52/61 (85.2%)	0.461
Emergency Medicine	2/10 (20.0%)	12/14 (85.7%)	0.003
OB-GYN	8/8 (100.0%)	6/12 (50.0%)	0.042*
Surgery	31/34 (91.2%)	34/35 (97.1%)	0.357

Internal medicine (N = 17/60, 28.3% in 2023, and N = 47/54, 87% in 2024) and pediatrics (N = 5/18, 27.8% in 2023 and N = 14/16, 87.5%) saw the greatest improvement (p <0.001). Family medicine (N = 8/14, 57.1% in 2023 and N = 15/16, 93.8% in 2024), emergency medicine (n/N = 2/10, 20% in 2023 and n/N = 12/14, 85.7% in 2024) also statistically increased. Ob-Gyn had a statistically significant decrease over the two years (n/N = 8/8, 100% to n/N = 6/12, 50% respectively).

Specialties were clustered into procedural predominant (emergency medicine, Ob-Gyn, and surgery) and non-procedural specialties (family medicine, internal medicine, pediatrics, and psychiatry). In the grouped cohorts for non-procedural specialties, significant improvement was seen from 2023 (n/N = 41/106, 38.7%) to 2024 (n/N = 83/94, 88.3%) with a p-value of <0.001. No significant change (p = 0.461) was seen in the procedural specialties from 2023 (n/N = 41/52, 78.8%) to 2024 (n/N = 52/61, 85.2%) as they maintained a higher level of performance in both years mostly driven by consistent performance in the surgery subgroup. Statistical differences were seen between the procedural and non-procedural subgroups in the “Quantitative Risk” and “Timeframe” domains in 2023 but not 2024 (Table 2).

**Table 2:** Clustered specialty subgroup analysis by domain for AY2023 and AY2024 IC = Informed Consent, AY = Academic Year * statistical significant difference between procedural and non-procedural groups

IC Domain	Year	Procedural n/N (%)	Non-Procedural n/N (%)	p-value
What	2023	52/52 (100.0%)	105/106 (99.1%)	1.000
	2024	61/61 (100.0%)	94/94 (100.0%)	1.000
Why	2023	52/52 (100.0%)	106/106 (100.0%)	1.000
	2024	61/61 (100.0%)	94/94 (100.0%)	1.000
How	2023	50/52 (96.2%)	97/106 (91.5%)	0.342
	2024	60/61 (98.4%)	91/94 (96.8%)	1.000
Benefit	2023	51/52 (98.1%)	105/106 (99.1%)	0.551
	2024	60/61 (98.4%)	93/94 (98.9%)	1.000
Qualitative Risk	2023	50/52 (96.2%)	101/106 (95.3%)	1.000
	2024	61/61 (100.0%)	92/94 (97.9%)	0.520
Quantitative Risk	2023	31/52 (59.6%)	25/106 (23.6%)	<0.001*
	2024	39/61 (63.9%)	54/94 (57.4%)	0.503
Alternatives	2023	42/52 (80.8%)	87/106 (82.1%)	0.830
	2024	53/61 (86.9%)	88/94 (93.6%)	0.165
Timeframe	2023	51/52 (98.1%)	53/106 (50.0%)	<0.001*
	2024	60/61 (98.4%)	92/94 (97.9%)	1.000

Facilitator group comparison

As an exploratory assessment of scoring patterns across facilitator groups, we compared minimum-standard pass rates between facilitators who graded in both years and facilitators who graded in only one year. Five facilitators graded in both years, scoring 20 OSCEs (12.6%) in AY2023 and 27 OSCEs (16.9%) in AY2024. No statistically significant difference in minimum-standard pass rates was observed between these facilitator groups. Because each encounter was scored by only one facilitator, these comparisons should not be interpreted as inter-rater reliability.

## Discussion

The multifaceted nature of IC, which varies based on procedural details, makes evaluating learner’s performance challenging. In this single-center TTR OSCE, a modified Spatz minimum-standard tool was feasible to implement and provided domain-level information about whether students addressed core consent-content elements during a standardized blood-transfusion consent encounter. This versatile tool can be used in both written IC [7] and in OSCEs. All specialties were included in this assessment, which highlights the broad potential of this rubric for educational use. 

Prior literature has demonstrated that graduating medical students and interns lack confidence and competence in obtaining IC despite its designation as a core EPA [4,5]. Our findings demonstrate that a structured, domain-based assessment tool can not only reliably evaluate IC performance in educational settings, but also detect meaningful improvements in specific domains over time. This highlights the need for the integration of explicit competency-based assessments that align with the EPA framework rather than relying on experiential learning alone [1,2].

In this minimum-standard OSCE assessment, AY2024 students more frequently met the overall minimum standard and more frequently addressed “Quantitative Risk,” “Alternatives,” and “Timeframes” compared with AY2023 students. These findings may reflect cohort differences, differences in implementation, changes in SP scripting, or differences in learner preparation. It reflects the utility of the tool in capturing changes in the curriculum implementation or student IC skill. This tool can be used to monitor progress of educational initiatives, such as increased focus on IC in various pre-clerkship and clerkship activities, to promote earlier exposure to EPA 11. Our results show the need for continued improvement in preparing students to explain quantitative risk in IC discussions. 

Students in procedural rotations demonstrated higher baseline proficiency in these specific domains, which accounts for the lack of statistically significant improvement in that subgroup. We hypothesize that students entering procedural specialties were more aware of the need to master IC skills from their experiences on rotations. This may explain the observed differences in the domains of quantitative risk and timeframe in 2023 and merits further study. 

Prior studies have identified interrater reliability as a major limitation in OSCE-based assessments, including variability among examiners with different backgrounds and training levels [8,9]. In our study, no training on tool usage was provided for facilitators after the initial AY2023 implementation, and facilitator-group pass rates did not differ significantly in exploratory comparisons. Our findings suggest that a well-designed tool may mitigate these concerns even when implemented by a large cohort of faculty and resident evaluators in the absence of formal rater training. The ease of use of this tool can be valuable to educators with limited access to expert graders. Future studies on student self-assessment or near-peer assessment could also expand the utility of this tool.

While multiple studies have been published regarding the importance of evaluating IC and student readiness, this modified minimum assessment tool may be useful as a practical educational monitoring instrument because it identifies specific consent-content elements that are omitted during observed encounters [4,10]. Used longitudinally and across multiple encounters, it could contribute to entrustment decisions in undergraduate or graduate medical education.

Limitations

This study has several limitations. This was a single-center study targeting fourth-year medical students during their mandatory TTR courses, and the findings may not generalize to other institutions, clinical contexts, or consent scenarios. While attempts are made to mimic a real patient encounter, obtaining IC in an OSCE setting is still an educational scenario. Minimal information was provided to students prior to the session, which could affect student performance. Some studies provided extensive information to students such as complication rates and other details [11]. In the real-life scenario, consenting providers are expected to recall or research this information to augment their discussions.

Blood-transfusion consent may differ from more complex procedural or surgical consents. In addition, AY2023 and AY2024 represented separate cohorts, and the assessment conditions were not fully identical because facilitator preparation differed across years. Therefore, year-to-year differences should be interpreted as observed cohort differences under similar but non-identical implementation conditions rather than as causal evidence of improved competence, curricular effect, or tool validity. Finally, because each recording was scored by only one facilitator, formal inter-rater reliability could not be assessed.

A recent study for an IC Assessment Scale (ICAS) [12] focuses primarily on the communication continuum in a simulated office visit for surgical consent. However, the content of the IC itself is not explicitly addressed. On the other hand, the modified Spatz tool addresses the knowledge components of IC without explicitly addressing the empathetic and communication competencies. Our SPs complete a separate empathy scale [13] to address the latter component. Both components are important and evaluated by educators in assessing entrustability for IC. 

## Conclusions

Medical students must graduate with the expected IC skills. The modified Spatz tool was feasible to implement. It provided a structured method for identifying whether students addressed core consent-content domains and can be applied to different procedures and interventions. The assessment tool is self-explanatory and requires minimal faculty training. Future studies should evaluate inter-rater reliability, gather external validity evidence, and examine whether repeated use of the tool across encounters can support entrustment decisions and curricular improvement.

## Appendices

**Table 3:** Modified abstraction tool and scoring approach SubQ - Sub-question Adapted from Spatz et al. [8] under the Creative Commons Attribution Non-Commercial 4.0 International (CC BY-NC 4.0) license and further modified by Marwaha et al. [6] also under the CC BY-NC 4.0 license.

Domain	Item	Intent	Response	Points
	Description of procedure			
Content	1. Is language describing what the procedure is (beyond the medical name) provided for the patient?	The name of the procedure (often written in medical jargon) should be restated in language that is readily understandable to most patients in order to facilitate communication and a common understanding about what procedure is being performed.	‘Yes’ ‘No’	1 0
SubQ	Name of the procedure only stated in medical terms Acronyms or abbreviations for which each letter is not defined (meaning, not spelled out AND defined elsewhere on the form) Name of a device without brief explanation of what it does Unexplained "possible" procedures Non-definitive statement of procedures (for example, AND/OR) Not provided		Multi-select	
Content	2. Is a description of how the procedure will be performed provided for the patient?	How the procedure will be performed should be described for the patient, as providing this information serves to inform the patient about what he or she can expect during the procedure.	‘Yes’ ‘No’	1 0
SubQ	Information about the procedure that does not explain HOW the procedure will be performed A list of procedure names, including procedures that may be performed Not provided		Multi-select	
	Rationale of procedure			
Content	3. Is the clinical rationale (condition-specific justification) for why the procedure will be performed provided?	The clinical rationale for the procedure should be clearly stated such that the patient is aware of why the procedure is being performed (that is, the intended impact of the procedure on the patient's condition/diagnosis)	‘Yes’ ‘No’	1 0
SubQ	Context not specified - Context does not map to any of the following: to diagnose/rule out, inform prognosis, treat, prevent, or execute the patient's request Condition not specified Language that states possible contexts, not explicitly defining a purpose An indirect outcome or benefit of the procedure on the patient's global health and wellbeing Acronyms or abbreviations used to state the patient's condition that are not previously spelled out Not provided		Multi-select	
	Patient-oriented benefit(s)			
Content	4. Is any patient-oriented benefit provided (intended impact on the patient’s health, longevity and/or quality of life)?	The patient-oriented benefit(s) of the procedure should inform the patient about the positive impact(s) that the procedure is anticipated to have on his or her health, longevity, and/or quality of life.	‘Yes’ ‘No’	1 0
SubQ	Language that refers only to the clinical rationale, without referring to a positive effect of the procedure on the patient Generic statement that the benefits were discussed with the patient Language that states possible benefits, not defining the primary (intended) benefit(s) Not provided		Multi-select	
	Probability of procedure-specifics risks			
Content	5. Is a quantitative probability provided for any procedure- specific risk?	A quantitative probability should be given so that the patient has a sense of the likelihood of a specific risk occurring. Providing the patient with a number enables him or her to determine whether the likelihood is acceptably low enough to proceed with the procedure.	‘Yes’ ‘No’	1 0
SubQ	Numeric prediction that states the risk is possible but provides no basis for comparison Generic statement that the clinician discussed the risks with the patient Not provided		Multi-select	
	6. Is a qualitative probability provided for any procedure- specific risk?	A qualitative probability should be given such that the patient has a sense of how often the procedure-specific risk occurs, albeit with less specificity than a quantitative probability (from the clinician or medical community's point of view). For some patients, a qualitative term may be more understandable and less intimidating than a numerical value. Patients may value the clinical or medical community's interpretation of the frequency of risk.	‘Yes’ ‘No’	1 0
SubQ	Generic terms that lack relativity/comparability Qualitative terms related to severity of risks (which do not indicate the likelihood of the risk occurring) Generic statement that the clinician discussed the risks with the patient Not provided		Multi-select	
	Alternative(s) to the procedures			
Content	7. Is any alternative provided for the patient?	Alternatives should be given such that the patient is aware of the options and he or she can make an informed decision. While the alternatives may have been discussed previously, restating the options in the informed consent document supports patient autonomy, providing the patient with an opportunity to ask questions and potentially seek a second option, if desired by the patient	‘Yes’ ‘No’	1 0
SubQ	Non-descriptive or generic statements about a patient's right to forego the procedure Generic statements about potential alternatives that are applicable across a range of surgical procedures Generic statement that the clinician discussed the alternatives with the patient No alternatives offered		Multi-select	
	Timeframe of the Procedure			
Content	8. Is a timeframe for the recommended procedure provided to the patient?	The patient or proxy for the patient should have enough time to thoughtfully consider the recommended procedure in context of its urgency.	‘Yes’ ‘No’	1 0
SubQ	Non-descript terms of timing used without clarifying timeframe Timeframe described was not accurate Not provided		Multi-select	
	Maximum Score			8

## References

[1] (2014). The core entrustable professional activities (EPAs) for entering residency. https://www.aamc.org/what-we-do/mission-areas/medical-education/cbme/core-epas.

[2] Lin YK, Liu KT, Chen CW, Lee WC, Lin CJ, Shi L, Tien YC (2019). How to effectively obtain informed consent in trauma patients: a systematic review. BMC Med Ethics.

[3] Chryssofos S, Glickman C, Mowdawalla C, Burden A, Xu YE (2025). Current practices, limitations, and recommendations for informed consent education in medical students and physicians: a scoping review. J Med Educ Curric Dev.

[4] Anderson TN, Aalami LR, Lee EW, Merrell SB, Sgroi MD, Lin DT, Lau JN (2020). Perception and confidence of medical students in informed consent: a core EPA. Surgery.

[5] Fhaolain SN, Griffin J, Rohan P, Keane KG, McLornan L (2024). Persistence of inadequate consent training for interns. Ir J Med Sci.

[6] Spatz ES, Suter LG, George E (2020). An instrument for assessing the quality of informed consent documents for elective procedures: development and testing. BMJ Open.

[7] Marwaha M, Bhalla R, Rao S, Chen C (2023). Minimum standard assessment of informed consent for internal medicine transition to residency program: a cohort study. Health Sci Rep.

[8] Schwartzman E, Hsu DI, Law AV, Chung EP (2011). Assessment of patient communication skills during OSCE: examining effectiveness of a training program in minimizing inter-grader variability. Patient Educ Couns.

[9] Mortsiefer A, Karger A, Rotthoff T, Raski B, Pentzek M (2017). Examiner characteristics and interrater reliability in a communication OSCE. Patient Educ Couns.

[10] Petravick ME, Marsh JL, Karam MD, Dirschl DR (2018). A survey on recent medical school graduate comfort with the level 1 milestones. J Surg Educ.

[11] Kempner S, Morgan H, Stern D (2016). Providing informed consent: a standardized case. MedEdPORTAL.

[12] Silva Gonçalves N, Morgado P, Collares CF, Pêgo JM (2025). Psychometric validation of the informed consent assessment scale using item response theory and factor analysis. Front Med (Lausanne).

[13] Terregino CA, Copeland HL, Sarfaty SC, Lantz-Gefroh V, Hoffmann-Longtin K (2019). Development of an empathy and clarity rating scale to measure the effect of medical improv on end-of-first-year OCSE performance: a pilot study. Med Educ Online.

